# Identification and Functional Analysis of V-ATPaseA and C Genes in *Hyphantria cunea*

**DOI:** 10.3390/insects15070515

**Published:** 2024-07-10

**Authors:** Xiaojie Wang, Dan Zhao, Qian Wang, Yanan Liu, Xiujun Lu, Wei Guo

**Affiliations:** 1 College of Plant Protection, Hebei Agricultural University, Baoding 071000, China; wxjwxj152@163.com (X.W.); zhbzhaodan@163.com (D.Z.); wangqian_711@126.com (Q.W.); 13331287884@126.com (Y.L.); guowei05@caas.cn (W.G.); 2 Graduate School of Chinese Academy of Agricultural Sciences, Beijing 100081, China

**Keywords:** *Hyphantria cune*, *HcV-ATPase A*, *HcV-ATPase C*, RNA interference, midgut structure

## Abstract

**Simple Summary:**

*Hyphantria cunea* is an important quarantine pest in the world; it is a crucial and pressing issue to seek new approaches to its management. In this study, V-ATPase genes are identified from *H. cunea* and their phylogenetic relationships are confirmed. Using histopathology analysis of larval after RNA interference (RNAi) treatment, this study has determined that *HcV-ATPase A* and *HcV-ATPase C* play crucial roles in the development of *H. cunea* by, affecting the growth and development of intestinal tissue. The *HcV-ATPase A* and *C* gene silencing performed increased lethality, which suggests that they could potentially serve as practicable RNAi targets. Future research on the function of *V-ATPase* may illuminate the mechanisms of vesiculation in insect intestinal wall cells, provide new target genes for pest control using molecular techniques, and optimize pest management strategies.

**Abstract:**

Vacuolar (H^+^)-ATPases (V-ATPases) are ATP-driven proton pumps that play multifaceted roles across various organisms. Despite their widespread significance, the functional implications of *V-ATPase* genes in *Hyphantria cunea*, an invasive forest pest with a global presence, have yet to be elucidated. In this study, two specific *V-ATPase* genes from *H. cunea* were identified and analyzed, namely *HcV-ATPase A* (accession number: OR217451) and *HcV-ATPase C* (accession number: OR217452). Phylogenetic analysis and multiple sequence alignment reveal that HcV-ATPase A shares the highest amino acid sequence similarity with SfV-ATPase A, while HcV-ATPase C is most similar to HaV-ATPase C. Spatiotemporal expression profiles, determined via RT-qPCR, demonstrate that both *HcV-ATPase A* and *HcV-ATPase C* are expressed throughout all larval developmental stages, with *HcV-ATPase A* predominantly expressed in the midgut and *HcV-ATPase C* showing high expression in the epidermis. RNA interference (RNAi) targeting of these genes significantly suppressed their expression by 62.7% and 71.0% 120 h post-injection, leading to halted larval growth and increased mortality rates of 61.7% and 46.7%, respectively. Further investigations using immunohistochemistry, hematoxylin and eosin (HE) staining, and transmission electron microscopy (TEM) revealed that gene silencing induced vesiculation and subsequent losses or sloughing of intestinal parietal cells, alongside an increase in the number of autophagic cells. Additionally, the silencing of *HcV-ATPase A* and *C* genes resulted in a reduced gut epidermal cell layer thickness and further increases in goblet cell numbers. Importantly, RNAi of *HcV-ATPase A* and *C* did not affect the expression levels of one another, suggesting independent functional pathways. This study provides foundational insights into the role of *V-ATPase* in *H. cunea* and identifies potential targets for the biocontrol of its larvae, contributing to the understanding of *V-ATPase* mechanisms and their application in pest management strategies.

## 1. Introduction

The fall webworm, *Hyphantria cunea*, is a polyphagous folivore pest originating from North America and Mexico, now widespread across the temperate regions of the Northern Hemisphere [1]. In China, *H. cunea* represents a significant quarantine pest in agriculture and forestry, with outbreaks occurring successively over the years and an expanding scope of damage [2]. Currently, chemical control is the primary method employed against *H. cunea* in agricultural production [3,4]. However, this approach often leads to the unintended killing of natural enemies, ecosystem disruption, the development of pest resistance, and environmental pollution due to pesticide residues. Since the discovery of RNAi in *Caenorhabditis elegans* [5], RNAi has been extensively utilized for gene function research and has emerged as a novel strategy for integrated pest management [4]. Therefore, identifying effective RNAi target genes is crucial for the successful application of RNAi-based pest management technologies.

The V-type ATPase was initially identified in the vacuolar membrane of yeast cells, leading to a series of detailed investigations into its structure and function [6,7]. V-ATPase contains V_1_ and V_o_ complexes, among which there are eight types of V_1_ complexes, including A and C. The V_o_ complex has different copy numbers in different organisms, which promote proton translocation through rotation mechanisms [8]. V-ATPase has been found to be ubiquitously present in insect epithelial cells in the midgut, Malpighian tubules, and salivary glands. The ATP hydrolysis activity of V-ATPase releases substantial energy, contributing to protein transport, receptor-mediated endocytosis, and pH regulation [9].

Several studies have indicated that V-ATPases have a significant impact on insect growth and development, including molting and reproduction. However, different subunits have various functions in different organisms. For example, the V ATPase A gene had been found to have insecticidal effects on *H. cunea*, but its mechanism of action is not known [10]. Transmission electron microscopy (TEM) revealed partially damaged brush borders in *Locusta migratoria* ds*LmV-ATPase A*-injected larvae [11]. *V-ATPase B*in *Periplaneta fuliginosa* resulted in nymphal molting defects characterized by the incomplete shedding of old cuticles, growth inhibition, and deformed, wrinkled thoracic and abdominal cuticles [12]. The cloning and subsequent silencing of the gene encoding the *V-ATPase H* subunit from *L. migratoria* led to mortality and various molting defects [13]. Co-ingestion of siRNAs specific to the *Helicoverpa armigera* coatomer β and *V-ATPase A* subunits resulted in gene co-silencing, reducing larval survival rates and weight gain [14]. Silencing the *HvV-ATPase A* and *HvV-ATPase E* genes significantly increased mortality in *Henosepilachna vigintioctopunctata* larvae [8]. Oral administration of a double-stranded RNA specific to the *V-ATPase A* subunit of *Tuta absoluta* led to a gradual decrease in gene expression from day 1 to 3, resulting in significant larval mortality [15]. When the *V-ATPase A* gene of *Apolygus lucorum* was silenced, larvae died, although adults did not [16]. Silencing the *V-ATPase A* and E genes in *A. lucorum* caused larval mortality rates ranging from 46% to 80% [17]. In *L. migratoria*, *LmV-ATPase-c* was indispensable for new cuticle formation during molting and played a crucial role in dsRNA escape from endosomes [18]. Silencing *LmV-ATPase-c* resulted in severe molting defects and high mortality rates. Histological staining and microscopic examination of abdominal cuticle sections revealed the absence of newly formed cuticles in larvae injected with ds*LmV-ATPase-c*. These findings suggest that the *V-ATPase A* and *C* subunits are critical targets in many insects, playing a vital role in their growth and development. However, the differences in the mechanism of action between *V-ATPase A* and *C* in RNAi responses remain to be elucidated.

Here, we hypothesize that *V-ATPases* are crucial for the development of the insect intestinal tract and have a lethal effect on *H. cunea*. The functional analysis of the *V-ATPase A* and *C* subunits in *H. cunea* was performed through RNAi. Verifying this hypothesis could advance research on a promising target for developing novel control strategies against this significant pest.

## 2. Materials and Methods

### 2.1. Insects

Eggs of *H. cunea* were obtained from the Insect Virus Research and Development Center, Institute of Forest Ecological Environment and Protection, Chinese Academy of Forestry Sciences. The larvae were reared on an artificial diet under conditions of 26 ± 1 °C, and 60% ± 5% RH (relative humidity), and a photoperiod of 16 h light/8 h dark.

### 2.2. Cloning and Validation of cDNA Seqences of HcV-ATPases

The cDNA sequences of *HcV-ATPases* were retrieved from the *H. cunea* transcriptome database. The full-length cDNA sequences were subsequently verified through PCR and nucleotide sequencing. Primers for this process were designed using DNAMAN 9.0 software (Table A1). Using the TaKaRa MiniBEST Universal RNA Extraction Kit (Takara, Japan) and MonAmp™ 2× MonHi-Fi Max Mix (Monad, Wuhan, China), RNA was extracted; we performed reverse transcription for cDNA, as well as PCR amplification. The PCR products were finally sequenced by Sangon Biotech (Shanghai, China).

### 2.3. Bioinformatics Analysis of HcV-ATPases

Multiple sequence alignment and sequence homology analyses were performed using NCBI BLASTp and ClustalX 2.0, respectively. Phylogenetic analysis of the V-ATPase subunits A and C, using sequences from different insects, was carried out with the neighbor-joining method in MEGA11. Molecular weight (MW) and isoelectric point [19] predictions were made using DNAMAN.

### 2.4. Expression Pattern of HcV-ATPases in Different Developmental Stages and Tissues

To analyze the stage-dependent expression of *HcV-ATPase A* and *C*, whole bodies of larvae from the 1st instar to pupae were collected. For the study of tissue-dependent expression, specific tissues including the head, foregut, midgut, hindgut, Malpighian tubules, epidemis, and fat body were dissected from 3rd-instar larvae. The experiment was conducted with three biological replicates, pooling three larvae per replicate. Total RNA extraction and first-strand cDNA synthesis were performed as described previously. The RT-qPCR reactions utilized SYBR Green qPCR Master Mix (UE, Suzhou, China) on a Bio-Rad system (Bio-Rad Laboratories, Hercules, CA, USA). Gene-specific primers for *HcV-ATPase A* and *HcV-ATPase C* were used, along with a specific primer pair for amplifying actin as a reference gene (Table A1).

### 2.5. Functional Analysis of HcV-ATPases by RNAi

To further investigate the functions of *HcV-ATPase A* and *C* in *H. cunea*, RNAi experiments were conducted. dsRNA for *HcV-ATPase A*, *HcV-ATPase C*, and *GFP* (accession number: KC896843) (as a control) were synthesized in vitro using the T7 RiboMAX™ Express RNAi System (Promega, Madison, WI, USA). Primers were designed using DNAMAN (Table A1). A microinjector was employed to inject 10 μg of dsRNA into the 3rd-instar larvae between the second and third abdominal segments. A total of 3 replicates were carried out for each dsRNA-treated group, consisting of 3 treatments and 20 replicates per treatment, respectively. Total RNA was prepared from three insects of each biological replicate, and the relative expression levels of *HcV-ATPase A*, *HcV-ATPase C*, and *actin* were determined by RT-qPCR. The phenotypes of the insects and the number of dead larvae were recorded to calculate the larval mortality rate. Data were analyzed using SPSS.

### 2.6. H&E Staining and Transmission Electron Microscopy of Midgut

Following RNAi targeting of*HcV-ATPase A*and *HcV-ATPase C*, it was observed that larvae ingested significantly less artificial feed compared to those treated with ds*GFP*. To examine the impact on gut development in *H. cunea* after RNAi against *HcV-ATPase A* or *C*, intestinal dissections were performed. Seventy-two hours post-dsRNA injection, the entire guts of the larvae were dissected in precooled phosphate-buffered saline (PBS, pH 7.2). Three biological replicates were established, each consisting of three larvae.

To further investigate the roles of *HcV-ATPase A* and *C* in the midgut, hematoxylin–eosin (H&E) staining was conducted. A total of 72 h after dsRNA injection, the midguts were dissected and fixed in 3% (*v*/*v*) glutaraldehyde in 0.2 M phosphate buffer (pH 7.2). Following embedding, sectioning, and staining processes, the specimens were examined and photographed using a Nikon Eclipse E100 microscope [11]. For ultrastructural analysis of the midgut epithelium, TEM was performed as previously described [20]. Samples were embedded in epoxy resin 812 for 2 h, sectioned to 60–80 nm thickness using an ultramicrotome, and observed with a Hitachi HT7800 transmission electron microscope.

### 2.7. Immunohistochemistry

Immunohistochemistry was utilized to analyze the expression levels of *HcV-ATPase A* and *C* post-RNAi. The sections were blocked with bovine serum albumin (BSA) and incubated with primary antibodies specific to HcV-ATPases. HRP-conjugated Rabbit IgG/FITC (Servicebio, Wuhan, China) served as the secondary antibody for fluorescence detection. Propidium iodide [19], a potent nucleic acid stain that emits red fluorescence upon binding to DNA, was used for nuclear staining, thus highlighting nuclei in red. Images were captured using a Nikon transmission electron microscope (HT7800/HT7700, Tokyo, Japan) with excitations at 465–495 nm for HRP Rabbit IgG/FITC and 330–380 nm for PI.

### 2.8. Statistical Analysis

Relative expression levels of *HcV-ATPase A* and *HcV-ATPase C* were calculated using the 2^−ΔΔCt^ method [21]. PCR results were normalized to the expression of the beta-actin housekeeping gene. The results of gene expression and larval mortality were tested by Tukey’s HSD one-way ANOVA and using SPSS 18.0 software.

## 3. Results

### 3.1. Identification and Characterization of HcV-ATPase A and C

**Figure 1:**
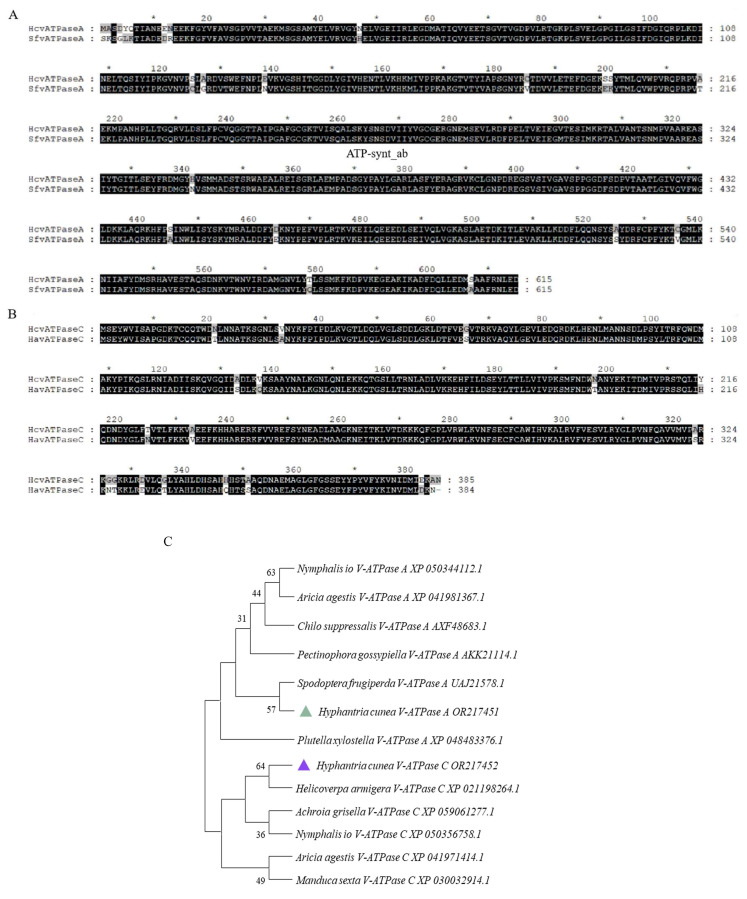
Sequence alignment and phylogenetic analysis of insect V-ATPase A and C. (**A**) Amino acid alignments of V-ATPase A from *S. frugiperda*. The ATP-synt_ab domain of HcV-ATPase A is underlined. (**B**) Amino acid alignments of V-ATPase C from *H. armigera*. (**C**) Amino acid sequence analysis and phylogenetic relationships of V-ATPase A and C from *H. cunea* and other animals. Phylogenetic trees of V-ATPases in insects and other organisms were constructed by using the neighbor-joining method. The deduced V-ATPase A amino acid of *H. cunea*was most closely related to the V-ATPase A protein of *S. frugiperda*. The deduced V-ATPase C amino acid of *H. cunea*was most closely related to the V-ATPase C protein of *H. armigera*. The green triangle represents the HcV-ATPase A protein amino acid. The blue triangle represents the HcV-ATPase C protein amino acid.

Upon searching the *H. cunea* transcriptome, we identified two genes encoding for the *V-ATPase A* and *V-ATPase C* subunits. The fulllength of these cDNA sequences were verified through PCR and nucleotide sequencing. The sequences were designated as *HcV-ATPase A* (GenBank accession number: OR217451) and *HcV-ATPase C* (GenBank accession number: OR217452). The full-length ORF of *HcV-ATPase A* comprises 1845 nucleotides, encoding 816 amino acids, while that of *HcV-ATPase C* includes 1158 nucleotides, encoding 385 amino acids. The calculated molecular weights (MWs) and isoelectric points (Chen, et al.) of the deduced proteins for HcV-ATPase A and C were 67.86 kDa and 43 kDa, and 4.79 and 8.64, respectively. The comparative phylogeny analysis based on the full-length amino acid sequences of HcV-ATPase A and C using the neighbor-joining method revealed that the V-ATPase A subunits from insects of different orders tended to cluster into a single group, with HcV-ATPase A closely related to *Spodoptera frugiperda* (GenBank accession number: UAJ21578.1) and exhibiting high similarity to V-ATPase A orthologs across various insect species (Figure 1). Similarly, HcV-ATPase C clustered with *H. armigera* (GenBank accession number: XP_021198264.1) and showed high similarity to V-ATPase C orthologs from different insect species (Figure 1). Based on sequence comparison, the consistency of amino acid sequences was 94.80%.

### 3.2. Expression Patterns of HcV-ATPase A and C

**Figure 2:**
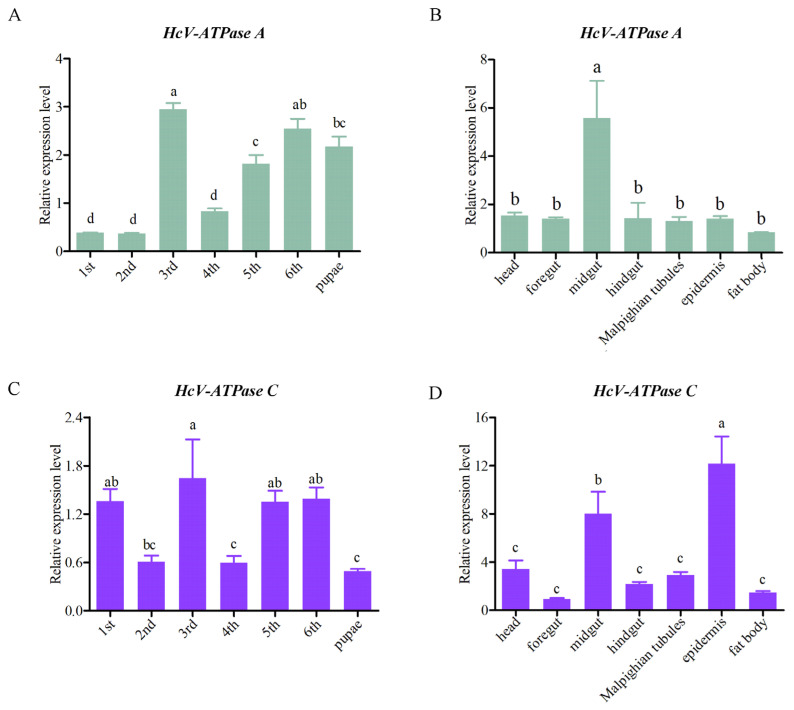
The expression patterns of *HcV-ATPase A* and *C* in different tissues and developmental stages. (**A**,**C**) The mRNA expression levels at different developmental stages on different days. (**B**,**D**) The mRNA expression levels in different tissues, with β-actin used as reference. The data are presented as mean ± S.D. of three independent biological replicates. As part of a one-way analysis of variance, significant differences between groups were determined by the Tukey’s test. The different letters (a–c) at the top of the bar graph represent the significant differences (n = 3) between different tissues and different developmental stages (*p* < 0.05).

To elucidate the expression patterns of *HcV-ATPase A* and *C* across various tissues and developmental stages, RT-qPCR analyses were conducted. These genes were found to be expressed in all tissues and developmental stages examined. Specifically, *HcV-ATPase A* showed peak expression in the midgut of third-instar larvae, while *HcV-ATPase C* was most abundantly expressed in the epidermis at the same stage, followed by the midgut (Figure 2). Notably, *HcV-ATPase A* demonstrated low expression levels during the first, second, and fourth-instar larvae stages, with higher expression observed in the third, fifth, and sixth-instar larvae and pupae. Conversely, *HcV-ATPase C* displayed a unique pattern of expression, with elevated levels in the first, third, fifth, and sixth-instar larvae, showing fluctuations across stages, and exhibited lower expression levels in the pupae. The data indicate that *HcV-ATPase A* and *C* genes may have distinct functions across different tissues and developmental stages of *H. cunea*.

### 3.3. Effect on H. cunea Survival after HcV-ATPase A and C RNAi

**Figure 3:**
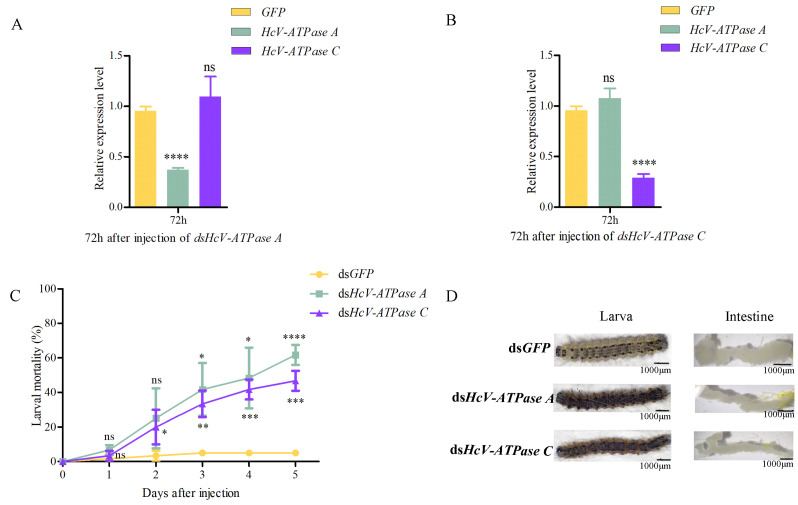
The effect of RNAi on *H. cunea* larvae and the expression levels of HcV-ATPase A and C. (**A**,**B**) The RT-qPCR method was used to detect the silencing efficiency of dsHcV-ATPase A and dsHcv-ATPase C. The transcription level of all target genes was normalized by the expression of the β-actin gene. (**C**) The survival rate of *H. cunea* after the injection of dsHcV-ATPase A or dsHcV-ATPase C. (**D**) The abnormal performance of *H. cunea* treated with dsHcV-ATPase A and dsHcV-ATPase C was compared with that of dsGFP. The third-instar larvae were treated with double-stranded RNA (dsRNA). The error line represents the standard deviation. The asterisk on the column indicates the significant difference between the control group and the dsRNA treatment group (* *p* > 0.05, ** *p* > 0.01, *** *p* > 0.0001, **** *p* > 0.00001). The data are expressed as the average of three biological replicates. As a part of the one-way ANOVA, Tukey’s test determined significant differences between groups (*p* < 0.01); ns means no significant difference, while * means significant difference.

To elucidate the biological roles of *HcV-ATPase A* and *C* in *H. cunea*, RNAi experiments were conducted. dsRNA targeting *HcV-ATPase A*, *HcV-ATPase C*, or a control ds*GFP* was injected into third-instar larvae. Relative to the control, the expression levels of *HcV-ATPase A* and *C* were significantly reduced by 62.7% and 71.0%, respectively, 72 h post-injection (Figure 3A,B). Notably, silencing one of these genes did not affect the expression of the other, indicating their independent action. Moreover, larval mortality rates increased to 61.7% and 46.7% for dsRNA-*HcV-ATPase A* and dsRNA-*HcV-ATPase C* treatments, respectively, after 120 h (Figure 3C). Post-injection observations included larval dehydration, rigidity, and darkening. Compared to the control group, the intestines of larvae subjected to dsRNA-*HcV-ATPase A* or dsRNA-*HcV-ATPase C* injections appeared thinner and more coiled (Figure 3D). These findings underscore the essential roles of *HcV-ATPase A* and *HcV-ATPase C* in *H. cunea* survival, highlighting them as critical targets for potential interventions.

### 3.4. Immunohistochemistry

**Figure 4:**
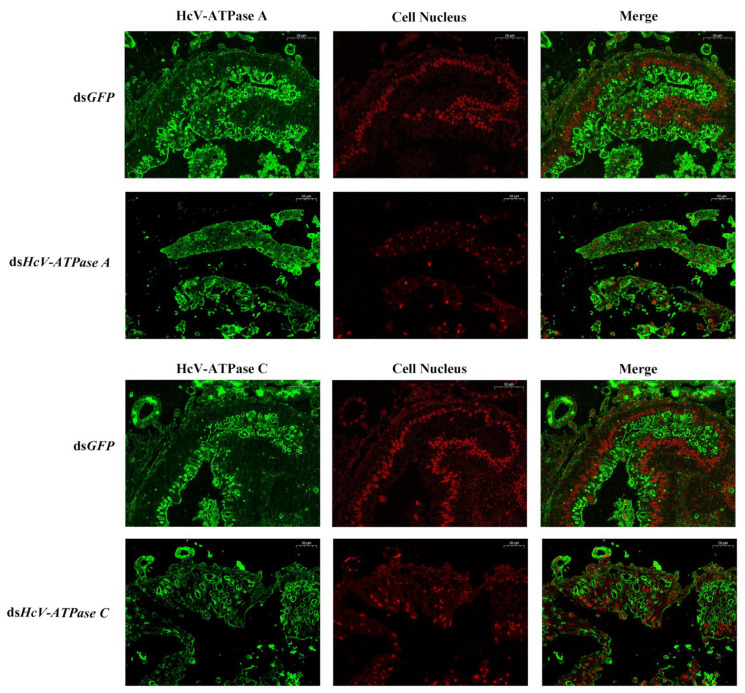
Immunofluorescence detection of *HcV-ATPase A* and *C* after RNAi. The detection of *HcV-ATPase A*/*C* using a specific antibody. The specific signal of *HcV-ATPase A*/*HcV-ATPase C* was detected after ds*GFP*, ds*HcV-ATPase A*, and ds*HcV-ATPase C* injection into third-instar larvae. The *HcV-ATPase A/C* protein is represented by green and the cell nucleus by red. The scale bar is 50 μm.

Immunohistochemistry was employed to investigate the expression and localization of *HcV-ATPase A* and *C*. In the intestinal wall cells of the midgut in *H. cunea* larvae, strong positive signals were observed in those treated with ds*GFP*, in contrast to the markedly weaker signals detected in larvae treated with ds*HcV-ATPase A* or ds*HcV-ATPase C*, using specific antibodies against *HcV-ATPase A* and *C* (Figure 4).

### 3.5. Effect of RNAi of HcV-ATPase A and C on H. cunea Midgut

**Figure 5:**
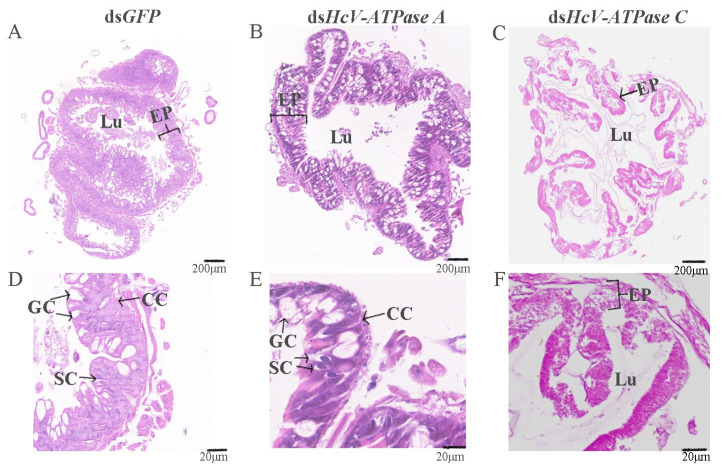
Histology analysis of midguts after injection with dsRNA-*GFP*/-*HcV-ATPase A*/-*HcV-ATPase C*.The third-instar larvae had ingested dsRNA-*GFP* (**A**,**D**), -*HcV-ATPase A* (**B**,**E**), and -*HcV-ATPase C* (**C**,**F**) for 3 days, shown by H&E staining. EP, epithelial. CC, columnar cell. GC, goblet cell. SC, stem cell. Lu, lumen. Scale bars: 200 μm and 20 μm.

H&E staining was utilized to examine the midguts of third-instar larvae following injection with dsRNA for *GFP*, *HcV-ATPase A*, and *C*. As depicted in Figure 5B,E, the midguts of larvae treated with ds*HcV-ATPase A* exhibited coiling and inward folding compared to the control, with the midgut lumen almost devoid of food. Given the midgut’s critical role in nutrient digestion and absorption, we prepared histological sections from dissected third-instar larvae midguts for H&E staining. This revealed a reduced number of columnar epithelial cells in the ds*HcV-ATPase A*-injected larvae, with these cells displaying inward folding and clustering. In the ds*HcV-ATPase A* treatment group, the apical region of some columnar cells (CCs) located in the posterior part of the midgut appeared slightly swollen and protruded into the intestinal lumen, suggesting a potential loss of contents. Additionally, there was a relative increase in the number of goblet cells (GCs), with changes in individual shape and an enlargement of the cavity. Cells near the intestinal lumen were observed to shed or disappear, while stem cells proliferated and differentiated. Conversely, in the ds*HcV-ATPase C* treatment group, the intestinal lining’s cell layer was disrupted and severely curled, with indiscernible cellular changes and a disordered intestinal wall structure (Figure 5C,F).

**Figure 6:**
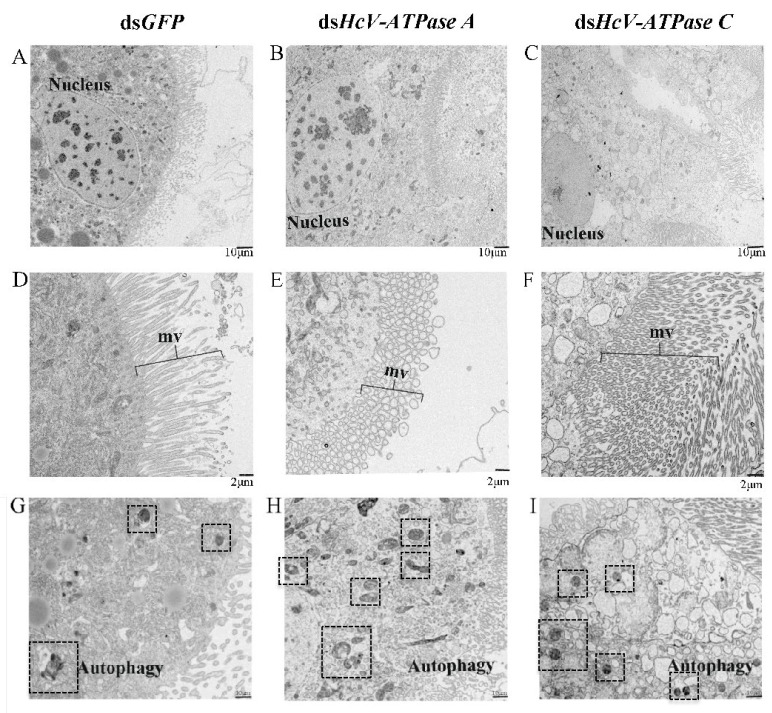
TEM analysis of midguts after injection with dsRNA-*GFP*/-*HcV-ATPase A*/-*HcV-ATPase C***.** The third-instar larvae had ingested dsRNA-*GFP*/-*HcV-ATPase A*/-*HcV-ATPase C* for 3 days, shown by TEM. (**A**,**D**) dsRNA-*GFP*. (**B**,**E**) dsRNA-*HcV-ATPase A*. (**C**,**F**) dsRNA-*HcV-ATPase C*. mv, microvillus. Scale bars: 10 μm (**A**–**C**,**G**–**I**) and 2 μm (**D**–**F**).

TEM further demonstrated that in larvae injected with ds*HcV-ATPase A*, nuclei divided and diminished, and numerous microvilli (mvs) underwent vesiculation. However, in the same ds*HcV-ATPase A*-injected larvae, nuclei shrank, a significant number of microvilli appeared broken and elongated, and the number of autophagic cells increased 28-fold compared with the control (Figure 6). In larvae treated with ds*HcV-ATPase C*, the cytoplasm of cell layers accumulated a high number of electron-lucent vesicles, the number of autophagic cells increased 2-fold, and the cell damage level was less than that with the ds*HcV-ATPase* A treatment. These results indicate that *HcV-ATPase A* and *HcV-ATPase C* are essential for the growth and development of columnar epithelial cells. The suppression of these genes hinders tissue development and reduces microvillus presence.

## 4. Discussion

Since its invasion of China in 1979, *H. cunea* has inflicted serious damage on China’s ecological environment, economic development, and biological safety [2]. The efficacy of current control methods for *H. cunea* is diminishing due to the development of insecticide resistance [22,23,24]. Identifying target genes that are specific to the pest or a narrow range of pests is crucial. Selecting suitable species-specific target genes is a pivotal initial step in devising effective RNAi-based control strategies. In this study, we analyzed the role of the multifunctional ATP-driven proton pump *V-ATPase* in *H. cunea* through the gene silencing of *V-ATPase* subunit *A* and *V-ATPase* subunit *C* using RNAi.

*V-ATPase* is a ubiquitous multi-subunit complex with different subunits, some of which are involved in ATP binding and hydrolysis, and some of which transport protons through the membrane in various chemometrics [25,26]. In insects, the structure, function, and regulation of V-ATPase have been widely studied, including in *H. cunea*. In this study, the cDNA sequences of *HcV-ATPase* subunits*A* and *C* were identified by searching the transcriptome of Oncomelania hupensis.

Amino acid residues essential for the activity or assembly of the *V-ATPase* complex have been identified in yeast and mouse models [26,27,28]. The *V-ATPase A* gene demonstrated the most robust and stable interference effect, highlighting the feasibility of targeting *V-ATPase A* as an alternative RNAi strategy for *H. cunea* control [10]. However, the physiological changes in *H. cunea* tissues induced by silencing the *V-ATPase A* gene have not been thoroughly investigated. This study thus focused on RNAi targeting of *V-ATPase A* and *V-ATPase C* genes, followed by histological analysis post-gene silencing. In mammals, *V-ATPase* subunits show various isoforms and are expressed in a cell or organelle-specific way to meet the different functions of *V-ATPase* [29]. RT-qPCR analysis in this study revealed high expression levels of *HcV-ATPase A* in the midgut, with *HcV-ATPase C* expression particularly elevated in the epidermis, followed by the midgut.

Lepidopteran species have generally shown resistance to RNAi, with several factors limiting RNAi efficiency, including dsRNA degradation, endosomal entrapment, malfunction of the core RNAi machinery, and restricted systemic spread [30]. Therefore, identifying highly efficient target genes is crucial for managing lepidopteran pests such as *H. cunea*. The foregut and hindgut develop from ectodermal invaginations during the embryonic period, whereas the midgut arises from the differentiation of endodermal mesenteric ligament cells. The midgut’s primary functions include cell secretion, food digestion, and nutrient absorption. The epidermis originates from a portion of undifferentiated embryonic ectodermal cells and serves as a protective barrier, facilitating the turnover from old to new epidermis. The high expression levels of *HcV-ATPase A* and *C* in these tissues suggest that these genes may have diverse functions in *H. cunea*. Tissue-specific expression patterns have been previously studied, with the highest *V-ATPase* expression reported in the midgut of *Bombyx mori* [19]. Consistent with these findings, our study identified the midgut as the site of the highest *HcV-ATPase A* gene expression, with *HcV-ATPase C* also showing elevated levels in the midgut, aligning with Chen H et al.‘s results. It is inferred that these genes are crucial for nutrient digestion and absorption in the midgut. Notably, *HcV-ATPase C* exhibited the highest expression in the epidermis, differing from *HcV-ATPase A’*s expression pattern. This multi-tissue distribution characteristic of the *V-ATPase* gene aligns with the findings of Wang J. et al. [31], underscoring the gene’s versatile roles across different tissues.

In this study, we further investigated the biological functions of *HcV-ATPase A* and *HcV-ATPase C* in *H. cunea* through dsRNA injections. Based on previous studies, it was found that 10 μg of dsRNA was suitable for RNAi silencing and the effect was good on the third day [32]. A total of three RNAi gene silencing experiments were carried out; the inhibition rate was between 55 and 71%, and the experimental error was small. On the first day post-injection of ds*HcV-ATPase A*and ds*HcV-ATPase C*, the expression of *HcV-ATPase C* and *HcV-ATPase A* increased significantly, respectively. This increase was based on *GFP*. The increase in abnormalities after the first day of gene silencing may be due to abnormal appearances caused by artificial injection techniques. Our findings indicate that both *HcV-ATPase A* and *C* are crucial for the survival of *H. cunea*. Similar developmental abnormalities have been observed following the silencing of other *V-ATPase* genes; for instance, silencing the *V-ATPase C* subunit gene in *L. migratoria* leads to developmental defects and mortality, while silencing the *V-ATPase A* gene in Apis mellifera results in larval but not adult mortality. These observations suggest that *V-ATPase* functions vary across different life stages. RNAi-mediated silencing of the *V-ATPase A* and *C* subunit genes resulted in developmental defects that were lethal to the larvae, underscoring the vital and conserved role of *V-ATPases* in insects. It is proposed that *V-ATPase* genes could serve as effective targets for controlling pests like *H. cunea*.

RNAi targeting of *HcV-ATPase A* and *C* also led to larval defects, notably a smaller intestine. This aligns with previous research reporting growth defects in *Diabrotica virgifera virgifera* and *H. armigera* following the silencing of *V-ATPase A* [33,34]. Given the midgut’s role in nutrient digestion and absorption, we dissected the alimentary canal to examine potential alterations resulting from RNAi targeting of *HcV-ATPase A* and *C*. We noted a marked absence of food in the midgut lumen of larvae treated with ds*HcV-ATPase A* and *C* compared with controls. Histological analysis revealed a reduced number of columnar epithelial cells, and in some areas, the brush border was completely absent after ds*HcV-ATPase A* treatments relative to the control. The brush border, formed by an array of microvilli extending into the ectoperitrophic space, is essential for intestine function, enhancing the surface area for nutrient absorption and the secretion of digestive enzymes and components of the peritrophic matrix [35]. In the midgut of *Manduca sexta* larvae, *V-ATPase* was shown to colocalize with and bind to actin filaments that support the microvilli of goblet cells [36]. Thus, the observed smaller intestine may result from a diminished number of columnar epithelial cells and reduced nutrient transport rates due to the compromised microvillar brush border following the knockdown of *HcV-ATPase A* and *C*.

Furthermore, specific accumulations of large electron-transparent vesicles were detected in the cytoplasm of midgut cells by histology and TEM analysis. The stimulation of gene silencing led to double the number of autophagy in cells, which were affected to varying degrees. In Drosophila, it has been proved that *V-ATPase A*plays a role in synaptic exocytosis of the nervous system [37]. Knockout of *V-ATPase A* also leads to the specific accumulation of synaptic vesicles [11,18,38]. We only took intestinal tissue of the test worm after 72 h and made three histopathological studies on the midgut, and the results of the three experiments were consistent. Intestinal tissue may be different at different times. This experiment needs to be further studied. In this study, we showed that *HcV-ATPase A* and *HcV-ATPase C*, similar to *V-ATPases* in other insects, were associated with vacuolation in goblet cells, columnar cells, autophagy cells, and microvilli. Unfortunately, we have not determined how changes following RNAi silencing affect these factors. Further studies are required to investigate the relationship between *HcV-ATPase A* and *HcV-ATPase C* and whether they act synergistically in the secretory digestion processes of the midgut.

## 5. Conclusions

This study has determined that *HcV-ATPase A* and *HcV-ATPase C* play crucial roles in the development of *H. cunea*. Our study indicates that *HcV-ATPase A* and *HcV-ATPase C* are key factors in midgut tissue, particularly during the development of intestinal parietal cells, thus being essential for the growth and development of *H. cunea*. Their high lethality when silenced suggests they could potentially serve as practicable RNAi targets. Future research on the function of *V-ATPase* may illuminate the mechanisms of vesiculation in insect intestinal wall cells, provide new target genes for pest control using molecular techniques, and optimize pest management strategies.

## Data Availability

All data generated or analyzed during this study are included in this published article.

## Appendix A

**Table A1:** Primers used in this study.

Primer Name	Sequence (5′-3′)	Application	Product Size (bp)
HcV-ATPase AF	CCGGAATTCATGGCGTCGGATTATCAG	cDNA verification	1845
HcV-ATPase AR	CCAAGCTTCTTAGTCCTCGAGGTTGCG		
HcV-ATPase CF	CCGGAATTCATGTCTGAATACTGGGTGA		1158
HcV-ATPase CR	CGCGTCGACCATTAGTTCGCCTTCTCG		
qHcV-ATPase AF	GTGATGTCAGCTGGGAATT	qRT-PCR	156
qHcV-ATPase AR	AGAAGGCGCGATGTATGT		
qHcV-ATPase CF	CTGATGACCTTGGTAAGCTTG		154
qHcV-ATPase CR	TATTTCGCCATGTCCCAT		
actinF	CTACCTCACGCCATTCTC		150
actinR	AGCTTCTCCTTGATGTCAC		
dsHcV-ATPase AF	TAATACGACTCACTATAGGGAGAAGATGCCGGCCAAC	RNAi	527
dsHcV-ATPase AR	TAATACGACTCACTATAGGCGTCCAGCTCGCTCGTAG		
dsHcV-ATPase CF	TAATACGACTCACTATAGGCCTACAATGCACTCAAGGGC		502
dsHcV-ATPase CR	TAATACGACTCACTATAGGGATCCAGGCGCAGAAACAC		
dsGFPF	TAATACGACTCACTATAGGCCACAAGTTCAGCGTGT		500
dsGFPR	TAATACGACTCACTATAGGAGTTCACCTTGATGCCG		

The underlined portion of the table is the restriction site.
